# Comparison of Amorphous and Crystalline Ni‐MOFs for Environmental Applications

**DOI:** 10.1002/open.202500373

**Published:** 2025-08-20

**Authors:** Viktorie Neubertová, Jaroslava Jarolímková, Stanislav Daniš, Ľuboš Vrtoch, Zdeňka Kolská

**Affiliations:** ^1^ Centre for Nanomaterials and Biotechnology Faculty of Science University of Jan Evangelista Purkyně Pasteurova 3632/15 Ústí nad Labem 400 96 Czech Republic; ^2^ Department of Physics Faculty of Science University of Jan Evangelista Purkyně Pasteurova 3632/15 Ústí nad Labem 400 96 Czech Republic; ^3^ Department of Chemistry Faculty of Science University of Jan Evangelista Purkyně Pasteurova 3632/15 Ústí nad Labem 400 96 Czech Republic

**Keywords:** amorphous, crystalline, gas sorption, metal‐organic frameworks, photocatalysis

## Abstract

Amorphous and crystalline nickel‐based metal‐organic frameworks (Ni‐MOFs) were prepared via a one‐pot synthesis at room temperature in methanol using 2‐methylimidazole as a ligand. The crystallinity was adjusted by varying the solvent volume, yielding an amorphous phase with higher surface area (≈242 m^2 ^g^−1^) and a crystalline form with reduced porosity (≈22 m^2 ^g^−1^). Comprehensive structural, morphological, and spectroscopic analyses confirmed distinct coordination environments, particle sizes and colloidal behaviors. Gas sorption measurements revealed enhanced CO_2_ uptake in the amorphous Ni‐MOF (≈9.5 cm^3 ^g^−1^) compared to the crystalline sample (≈3.4 cm^3 ^g^−1^), consistent with its greater pore volume and surface area. Photocatalytic degradation of methyl orange under 365 nm UV irradiation demonstrated faster activity for the amorphous material, with a pseudo‐first‐order rate constant of 0.0157 min^−1^ versus 0.0035 min^−1^ for the crystalline sample. These findings suggest that structural features such as higher surface area, pore volume, and possible disorder contribute to the improved gas sorption and photocatalytic response. The use of mild reaction conditions and a single solvent system offers a straightforward and energy‐efficient approach for preparing functional MOFs with tunable crystallinity, applicable in environmental remediation contexts.

## Graphical abstract

One‐pot synthesis of amorphous and crystalline Ni‐MOFs enables control of crystallinity by solvent volume. The amorphous material exhibits enhanced CO_2_ uptake and faster UV‐driven photocatalytic degradation of methyl orange under mild conditions.

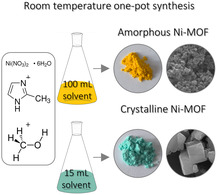

## 1 Introduction

The growing demand for efficient and environmentally responsible materials for energy and pollution control has placed metal‐organic frameworks (MOFs) among the functional materials researched over the past two decades.^[^ ^1^ ^,^ ^2^ ^]^ MOFs are coordination compounds formed from metal ions and organic linkers. They generally exhibit structural variability, large specific surface area, and adjustable porosity.^[^ ^3^, ^4^ ^–^ ^5^ ^]^ These frameworks can be found in either amorphous or crystalline phases, each having unique features and possible applications.^[^ ^6^ ^,^ ^7^ ^]^ Imidazolate‐based MOFs are widely studied for environmental applications such as gas sorption, pollutant degradation, and catalysis with frameworks based on zinc or cobalt being the most frequently investigated.^[^ ^8^, ^9^, ^10^, ^11^ ^–^ ^12^ ^]^ In contrast, nickel‐based MOFs (Ni‐MOFs) containing imidazolate ligands have received relatively less attention, despite nickel's advantageous catalytic properties and electronic configurations.^[^ ^13^ ^,^ ^14^ ^]^ From the perspective of environmental applications, the development of MOF‐based materials should also consider the sustainability of their preparation. Many reported methods for preparing Ni‐MOFs using 2‐methylimidazole (2‐MIM), which typically yield crystalline structures, rely on solvothermal procedures, increasing energy consumption.^[^ ^15^, ^16^, ^17^, ^18^, ^19^, ^20^, ^21^ ^–^ ^22^ ^]^ Some studies have also shown that the solvothermal method can lead to the formation of amorphous phases of these materials.^[^ ^23^ ^,^ ^24^ ^]^

One‐pot synthesis is a less commonly reported method for the preparation of imidazolate Ni‐MOFs. However, several studies have explored its potential. In one report, crystalline Ni‐MOFs were prepared in distilled water at room temperature (RT) using different reactant ratios, yielding green powders with varying crystallite sizes that were subsequently evaluated for energy storage applications.^[^ ^25^ ^]^ Another study by Tsai et al.^[^ ^26^ ^]^ investigated various imidazole‐based MOFs, including a nickel‐containing sample synthesized by mixing reactants in methanol at 60 °C for 1 h. Based on the broad diffraction features in the X‐ray diffraction pattern, the resulting material was identified as amorphous. This sample also exhibited strong UV–vis absorption properties, suggesting potential applicability in light‐driven processes.

While crystalline MOFs are more studied due to their well‐defined porosity and long‐range order, amorphous analogs are gaining increasing attention for their distinct properties. These disordered materials often feature higher densities of structural defects, improved surface accessibility, and enhanced diffusion characteristics, which can be beneficial for applications such as adsorption and photocatalysis.^[^ ^27^, ^28^ ^–^ ^29^ ^]^ Nevertheless, systematic comparisons between amorphous and crystalline forms remain limited, particularly for nickel‐based imidazolate frameworks synthesized under mild and environmentally friendly conditions.

Following previous studies, we present a simple RT synthesis of amorphous and crystalline Ni‐MOFs using 2‐MIM as the organic linker. By varying only the solvent volume, two distinct phases were obtained under mild and sustainable conditions. Methanol was used as the reaction medium due to its low toxicity and environmental compatibility compared to other organic solvents.^[^ ^30^ ^]^ The structural, morphological, and functional properties of the materials were studied and compared to evaluate how crystallinity affects their behavior. In addition, CO_2_ adsorption and photocatalytic degradation of methyl orange (MO) under UV light were investigated. MO was selected as a model azo dye due to its widespread use in dyeing industries and its common application in photocatalytic degradation studies.^[^ ^31^ ^]^ These findings contribute to the growing interest in sustainable MOF synthesis and highlight the potential of both amorphous and crystalline materials in environmental applications.

## 2 Results and Discussion

**Figure 1:**
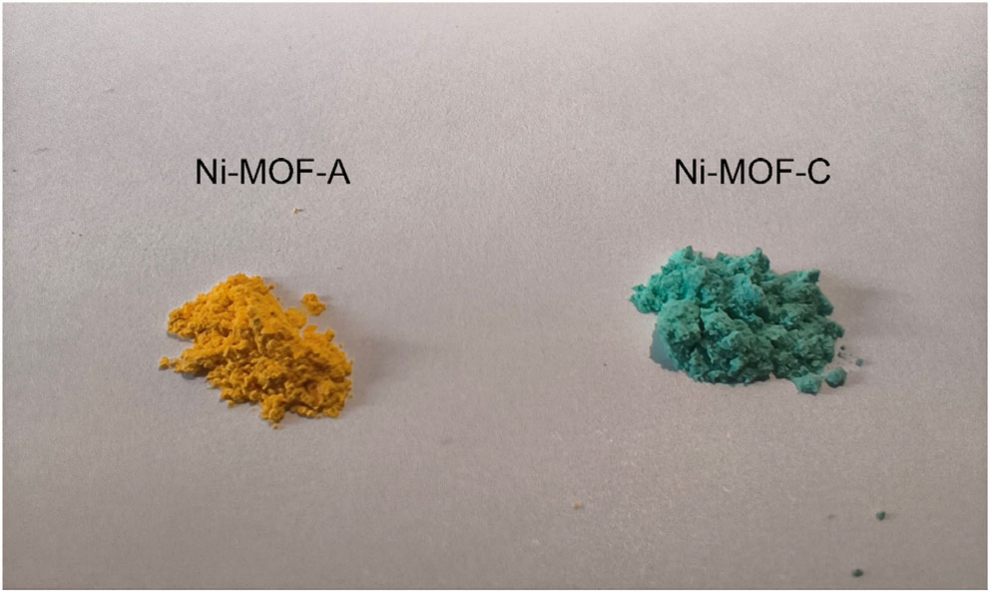
Photograph of Ni‐MOF‐A (yellow) and Ni‐MOF‐C (green) powders after drying.

During the preparation of Ni‐MOF‐A, a yellow precipitate began to form within the first hour, and upon standing overnight, the color intensified and the dried powder was bright yellow in color. As for Ni‐MOF‐C, during the synthesis, the solution started to become cloudy with green color after a longer time than that of Ni‐MOF‐A and the dried powder had a pale green color. A photograph of the resulting fine powder is shown in **Figure** **1**.

**Figure 2:**
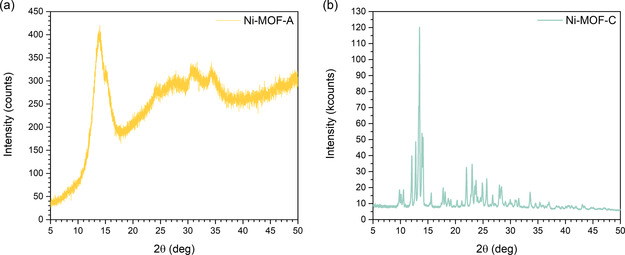
Powder XRD patterns of a) Ni‐MOF‐A and b) Ni‐MOF‐C.

The structural properties of Ni‐MOF‐A and Ni‐MOF‐C were investigated by powder XRD represented in **Figure** **2**. The diffraction pattern of Ni‐MOF‐A revealed a nearly amorphous structure, characterized by a broad feature without sharp Bragg reflections (Figure 2a). This indicates the absence of long‐range order and confirms the disordered nature of the framework. Such broad XRD features are typical for amorphous MOFs, where rapid nucleation, solvent effects, or synthesis conditions prevent the development of extended crystallinity.^[^ ^32^ ^]^ Similar behavior has been reported for amorphous zeolitic imidazolate frameworks (ZIFs), where the collapse of the crystalline network upon thermal treatment leads to the loss of Bragg peaks and the appearance of broad diffuse scattering.^[^ ^33^ ^]^ Additionally, Addai et al. reported the formation of amorphous Ni‐MOFs synthesized hydrothermally, where the XRD patterns displayed similar broad features.^[^ ^34^ ^]^ In contrast, the amorphous Ni‐MOF‐A in this study was synthesized via a simple one‐pot method at RT, without the need for hydrothermal conditions, demonstrating an accessible and energy‐efficient approach to amorphous MOF preparation.

The mean size of coherently diffracting domains (crystallite size) was estimated using the Scherrer equation, (Equation 1)

(1) $$D=\frac{K\lambda }{\beta \cos\theta }$$

where *D* is the crystallite size, *K* is the shape factor (taken as 0.9), *λ* is the X‐ray wavelength, *β* is the full width at half maximum (FWHM) in radians, and *θ* is the Bragg angle. Pseudo‐Voigt functions were fitted to the diffraction region near 14° 2*θ*, yielding an average crystallite size of ≈3.8 ± 0.2 nm for Ni‐MOF‐A.

The XRD pattern of Ni‐MOF‐C (Figure 2b) exhibited multiple sharp diffraction peaks at 2*θ* values of ≈9.8°, 10.5°, 13.2°, 17.8°, 22.0°, 23.4°, 24.9°, 25.7°, and 28.2°, confirming the formation of a well‐crystallized material. While the observed peaks confirm the formation of a crystalline nickel‐based framework, their positions and intensities differ from those reported for Ni‐MOFs synthesized via sonochemical method.^[^ ^35^ ^]^ This variation may result from the one‐pot RT synthesis used in this study, which could favor the formation of a distinct framework topology. Rietveld refinement using the Thomson‐Cox‐Hastings function yielded an isotropic crystallite size of 91 ± 3 nm, indicating the presence of well‐developed crystalline domains. Interplanar d‐spacings calculated using Bragg's law ranged from 9.02  to 3.16 Å, consistent with extended coordination frameworks. The high number of sharp reflections and their irregular spacing suggest a low‐symmetry structure, most likely triclinic. Such symmetry is frequently observed in flexible MOFs assembled from small imidazolate ligands under mild synthetic conditions. For instance, ZIF‐4 crystallizes in the triclinic system under similar parameters.^[^ ^36^ ^,^ ^37^ ^]^ Other nickel‐based coordination frameworks have also been reported to adopt monoclinic or triclinic symmetries depending on the synthetic method and solvent environment.^[^ ^38^ ^,^ ^39^ ^]^

**Figure 3:**
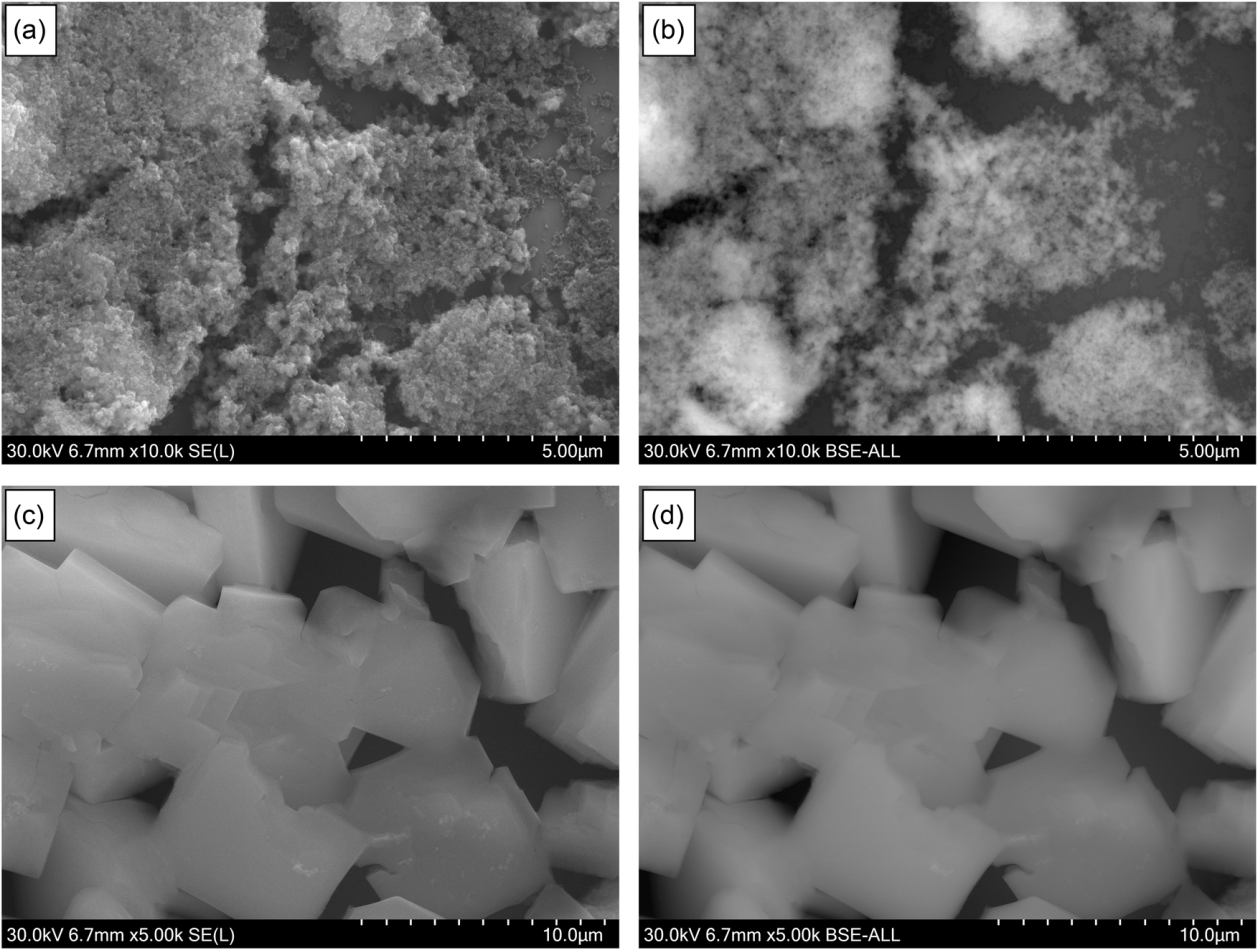
SEM images of Ni‐MOF samples: a) SE image of Ni‐MOF‐A, b) BSE image of Ni‐MOF‐A, c) SE image of Ni‐MOF‐C, and d) BSE image of Ni‐MOF‐C.

**Figure** **3** presents SEM images of the Ni‐MOF samples captured using both SE and BSE detectors to evaluate their morphological and compositional characteristics. Figure 3a shows the morphology of Ni‐MOF‐A captured using secondary electrons, highlighting its irregular and aggregated particle structure with no visible crystallite facets or boundaries, consistent with an amorphous material. The BSE image (Figure 3b) provides compositional contrast based on atomic number, indicating local variations in nickel distribution within the Ni‐MOF‐A sample. Both SE and BSE imaging consistently point to the amorphous character of the Ni‐MOF‐A. On the other hand, Ni‐MOF‐C displays compact particles with well‐defined edges and faceted surfaces in the SE image (Figure 3c), consistent with crystalline growth. The corresponding BSE image (Figure 3d) shows relatively uniform contrast, without visible phase separation or compositional inhomogeneity. These observations align with the XRD results and further confirm the crystalline nature of the sample.

EDX analysis confirmed the elemental composition of both Ni‐MOF‐A and Ni‐MOF‐C. The amorphous sample exhibited a higher proportion of carbon and a lower nickel content, while the crystalline phase showed a comparatively increased metal content and reduced organic fraction, which corresponds to its denser and more ordered structure. The nitrogen and oxygen values were comparable in both materials. However, due to the naturally low X‐ray yield of light elements such as carbon, nitrogen, and oxygen, and the difficulty in accurately quantifying them in matrices rich in organic matter, the uncertainties associated with these elements are naturally higher. Despite these limitations, the observed elemental trends clearly support the interpretation of Ni‐MOF‐A as a ligand‐rich amorphous phase and Ni‐MOF‐C as a more metal‐dense crystalline form. The quantitative EDX values along with their estimated uncertainties are summarized in **Table** **1**.

**Table 1:** Normalized EDX composition ( wt%) for Ni‐MOF‐A and Ni‐MOF‐C.

Element	Ni‐MOF‐A [ wt%] ±a)	Ni‐MOF‐C [ wt%] ±a)
C	62.4 ± 19.9	56.0 ± 12.5
N	21.6 ± 11.5	24.5 ± 7.9
O	10.8 ± 5.7	10.8 ± 3.7
Ni	5.3 ± 0.7	8.7 ± 0.8

^a)^ Uncertainties are derived from original 3*σ* values, adjusted for presentation clarity. Light‐element values (C, N, and O) are associated with greater variability due to limitations of EDX detection.

**Figure 4:**
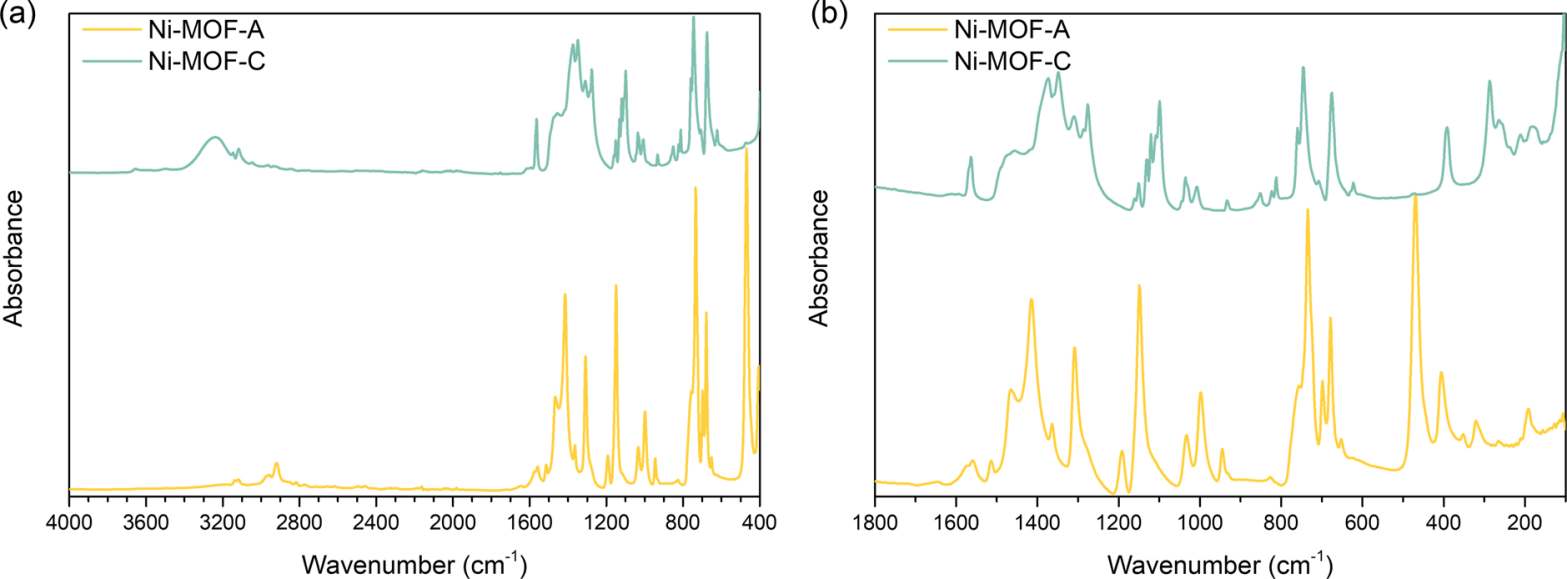
FTIR spectra of the samples Ni‐MOF‐A and Ni‐MOF‐C in the a) 4000–400 cm^−^^1^ region and b) 1800–100 cm^−1^ region.

The FTIR spectra of Ni‐MOF‐A and Ni‐MOF‐C (**Figure** **4**) exhibit distinct differences that reflect the coordination environment and degree of structural order in the amorphous and crystalline phases. Figure 4a presents FTIR spectra of both samples in the 4000–400 region. In the N–H stretching region, Ni‐MOF‐C displays a broad absorption band between 3373 and 3239 cm^−1^, commonly attributed to hydrogen‐bonded N–H vibrations of imidazole‐based ligands.^[^ ^24^ ^]^ The width and shape of this band suggest stronger and more uniform interactions between coordinated nitrogen atoms in the crystalline phase. In contrast, Ni‐MOF‐A exhibits narrower peaks within the 3239–3117 cm^−1^ range, which are consistent with less hydrogen bonding and a greater diversity of local environments, typical of amorphous coordination networks.^[^ ^22^ ^,^ ^24^ ^,^ ^40^ ^]^ Both samples show features in the aliphatic and aromatic C–H stretching region (3050–2800 cm^−1^). Ni‐MOF‐A presents absorptions at 2958, 2922, and 2854 cm^−1^, while Ni‐MOF‐C exhibits peaks at 2924 and 2854 cm^−1^, all assigned to methyl and methylene C–H stretches of 2‐MIM. Slight differences in intensity and separation suggest increased uniformity in the crystalline sample.

In a fingerprint region, a distinct band at 1618 cm^−1^ in Ni‐MOF‐C and 1606 cm^−1^ in Ni‐MOF‐A is attributed to the C=N stretching vibration of the imidazole ring. The upshift and narrowing in Ni‐MOF‐C indicate more consistent Ni–N coordination across the framework. Additional aromatic C–C and C–N stretching modes are observed between 1559 and 1349 cm^−1^ in both materials, but they appear sharper and more separated in the crystalline phase. In the region 1300–700 cm^−1^, Ni‐MOF‐C exhibits clearly resolved bands at 1243, 1171, 1111, and 1042 cm^−1^, corresponding to ring deformation and C–H bending modes. In contrast, Ni‐MOF‐A shows broader, overlapping features, consistent with a wider range of vibrational environments and structural disorder. Additional bands at 1012, 957, 914, 883, and 821 cm^−1^ correspond to out‐of‐plane bending vibrations of the imidazole ring. These also exhibit greater sharpness and regularity in the crystalline sample.^[^ ^41^ ^]^ In the low‐frequency region, peaks at 753 and 691 cm^−1^ are assigned to skeletal ring vibrations. Ni‐MOF‐C again presents more defined features, consistent with its higher symmetry and phase purity.

The far‐infrared spectra (400–100 cm^−1^) in Figure 4b offer further evidence of structural divergence. Ni‐MOF‐C shows multiple well‐defined peaks attributed to Ni–N stretching and metal‐ligand framework modes, supporting the presence of an ordered coordination network. In contrast, Ni‐MOF‐A exhibits broader and less resolved bands in this region, consistent with disordered Ni–ligand environments.

**Figure 5:**
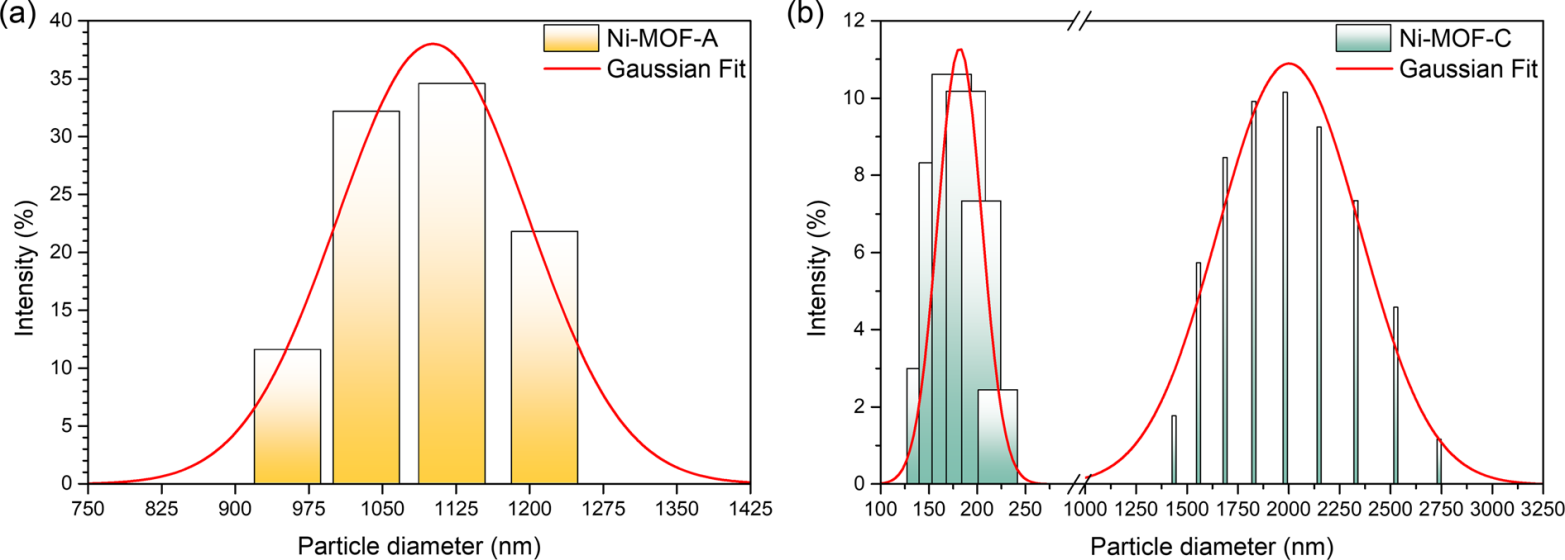
Particle diameter distribution of the a) Ni‐MOF‐A and b) Ni‐MOF‐C samples.

DLS measurements showed hydrodynamic diameters of 1410 ± 79 nm for Ni‐MOF‐A and 966 ± 71 nm for Ni‐MOF‐C, reflecting their distinct structural characteristics. The larger size of Ni‐MOF‐A is attributed to its disordered and porous nature, which promotes solvent penetration and aggregation in suspension.^[^ ^42^ ^]^ In contrast, the compact and ordered structure of Ni‐MOF‐C limits solvent interaction, resulting in a smaller hydrodynamic diameter.^[^ ^43^ ^]^ This behavior aligns with typical differences between amorphous and crystalline materials, where amorphous phases generally exhibit larger particle sizes due to lower packing density and greater structural flexibility. Also, **Figure** **5** shows the particle size distributions of Ni‐MOF‐A and Ni‐MOF‐C. The polydispersity index (PDI) was higher for Ni‐MOF‐A (41.7%) than for Ni‐MOF‐C (28.2%), indicating broader size distribution and lower structural uniformity. This reflects the inherent disorder and aggregation tendency of amorphous materials.^[^ ^44^ ^]^ In contrast, the lower PDI of Ni‐MOF‐C is consistent with its ordered structure, which favors uniform particle formation.^[^ ^45^ ^]^ The bimodal distribution observed for Ni‐MOF‐C suggests partial aggregation of individual crystallites in aqueous suspension.

Zeta potential measurements further highlight the differences in colloidal stability between the samples. Ni‐MOF‐A exhibited a value of 10.6 ± 2.1 mV, while Ni‐MOF‐C reached 34.4 ± 0.4 mV. The lower and more variable zeta potential of Ni‐MOF‐A suggests reduced electrostatic repulsion and, consequently, lower colloidal stability. This is likely due to its disordered surface, where non‐uniform charge distribution leads to irregular repulsive forces and promotes aggregation. The variability also implies the presence of heterogeneous surface charge sites. In contrast, the higher and more consistent zeta potential of Ni‐MOF‐C indicates strong electrostatic repulsion between particles and greater suspension stability, consistent with a well‐organized surface structure.^[^ ^46^ ^]^ The elevated zeta potential of Ni‐MOF‐C may also reflect a higher density of positively charged surface groups, potentially resulting from differences in the coordination environment and ligand orientation around Ni centers.^[^ ^47^ ^,^ ^48^ ^]^

**Figure 6:**
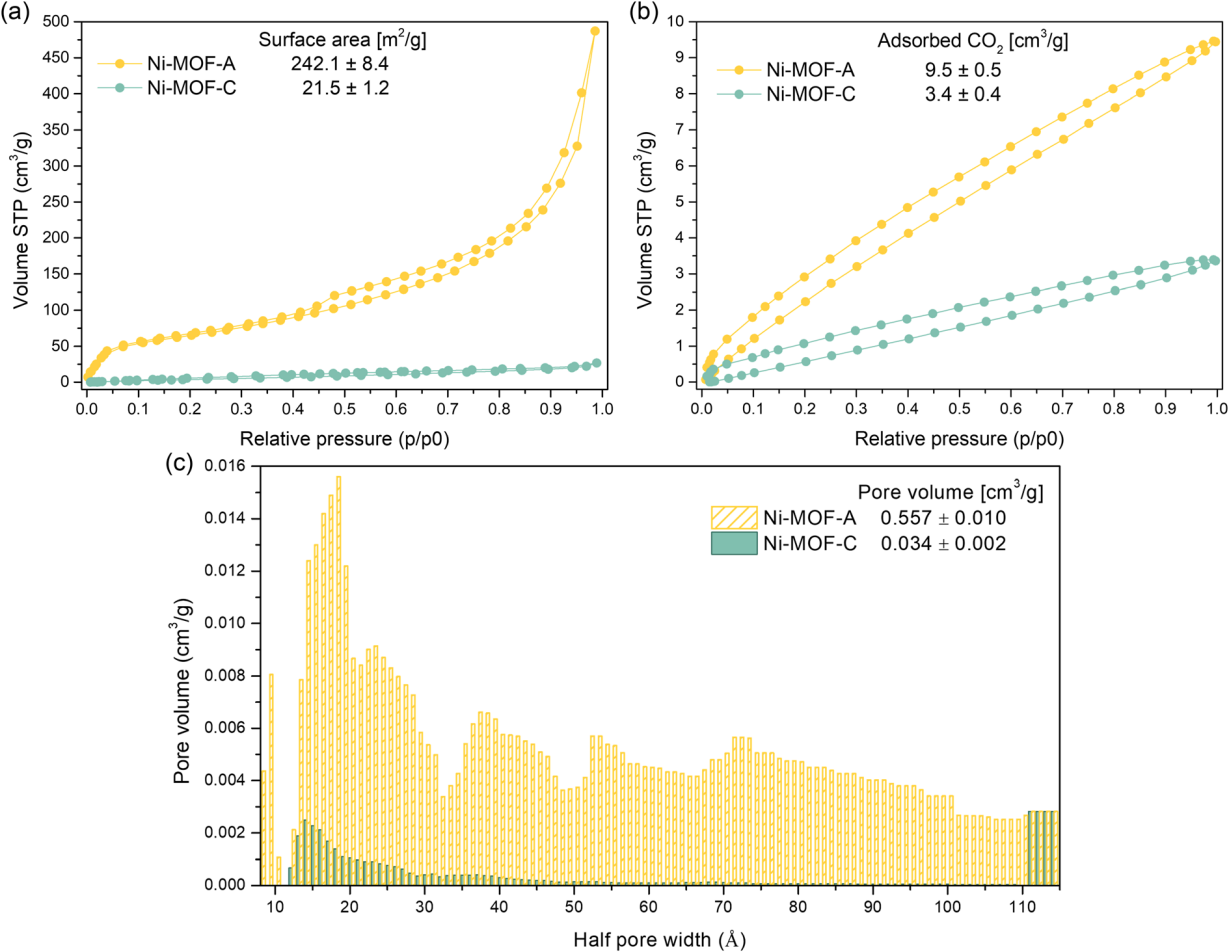
BET analysis of Ni‐MOF‐A and Ni‐MOF‐C a) N_2_ adsorption–desorption isotherms at 77 K, b) CO_2_ adsorption–desorption isotherms at 0 °C, and c) pore size histograms.

Surface area, pore volume, and pore size distributions, key parameters for gas sorption and catalytic applications, were evaluated and are presented in **Figure** **6**. The nitrogen adsorption–desorption isotherms at 77 K (Figure 6a) reveal a clear contrast between the amorphous and crystalline phases. Ni‐MOF‐A exhibits a significantly higher BET surface area of 242.1 ± 8.4 m^2 ^g^−1^, as evidenced by greater nitrogen uptake across the entire relative pressure range (*P*/*P*_0_). This is consistent with the highly porous and irregular structure characteristic of amorphous materials.^[^ ^6^ ^]^ In contrast, Ni‐MOF‐C shows a much lower BET surface area of 21.5 ± 1.2 m^2 ^g^−1^, indicating limited porosity. These results align with findings by Rajan et al. on Ni‐MOFs synthesized via solvothermal routes.^[^ ^24^ ^]^

The observed differences in surface area and pore volume are also reflected in CO_2_ sorption behavior (Figure 6b). Ni‐MOF‐A demonstrated a CO_2_ uptake of 9.5 ± 0.5 cm^3 ^g^−1^, nearly 3 times higher than that of Ni‐MOF‐C (3.4 ± 0.4 cm^3 ^g^−1^). To the best of our knowledge, this is the first direct comparison of CO_2_ adsorption between amorphous and crystalline Ni‐MOFs, indicating their gas sorption potential.

Finally, the pore size distributions and corresponding pore volumes are presented in Figure 6c. Ni‐MOF‐A exhibits a higher pore volume of 0.557 ± 0.010 cm^3^ g^−1^, compared to 0.034 ± 0.002 cm^3 ^g^−1^ for Ni‐MOF‐C, showing a greater abundance of accessible pores in the amorphous structure. The pore size distribution of Ni‐MOF‐A spans a wider range, extending from small mesopores (≈20 Å) to larger mesopores and macropores exceeding 100 Å. This broad distribution suggests a complex and heterogeneous pore network. In contrast, Ni‐MOF‐C displays a much narrower pore size range, consistent with its more compact and ordered crystalline framework.

**Figure 7:**
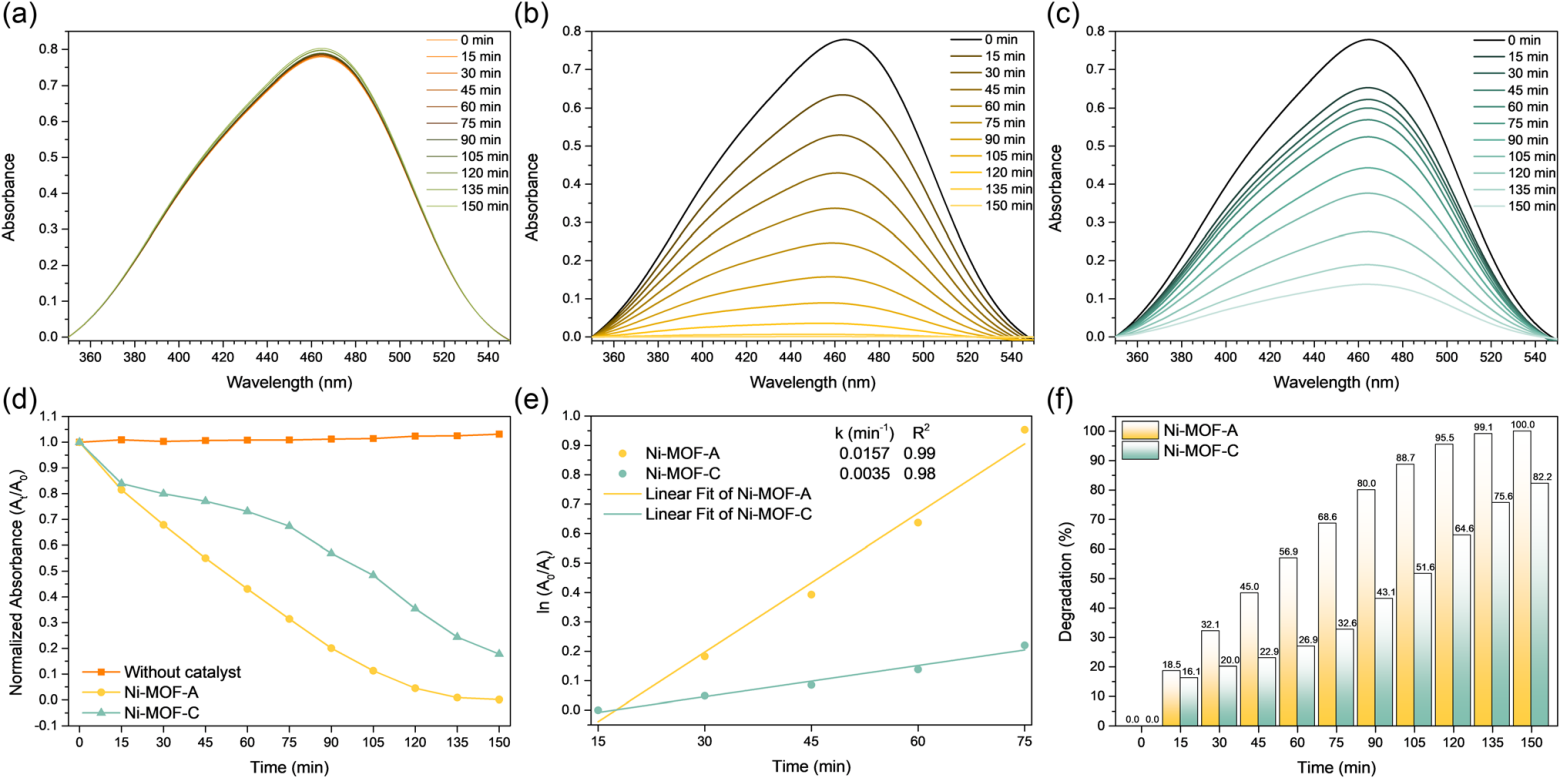
Photodegradation of MO under UV light (*λ *= 365 nm): a) UV‐Vis spectra for blank, b) UV‐Vis spectra for Ni‐MOF‐A, c) UV‐Vis spectra for Ni‐MOF‐C, d) normalized absorbance (*A*_t_/*A*_0_) at 464 nm, e) pseudo‐first‐order kinetics with *k*, and f) degradation efficiency over time for Ni‐MOF‐A and Ni‐MOF‐C.

**Figure** **7** presents the UV‐Vis spectral evolution during photocatalytic degradation of MO under 365 nm UV light. In the control experiment without catalyst (Figure 7a), the characteristic absorbance peak at 464 nm remained essentially unchanged over the 150 min exposure. A slight increase in absorbance was observed at some time points, likely due to thermal fluctuations or spectrophotometric baseline drift, rather than any actual chemical transformation. These minor variations confirm the photostability of MO under the applied conditions and validate that degradation observed in the presence of catalysts is photocatalytic in origin. When Ni‐MOF‐A was added (Figure 7b), a rapid and continuous decrease in the 464 nm absorbance was observed, reaching zero by the end of the experiment. In contrast, Ni‐MOF‐C (Figure 7c) exhibited slower degradation, with a residual peak still visible at 150 min. This trend is further supported by normalized absorbance plots (*A*_t_/*A*_0_, Figure 7d), where Ni‐MOF‐A displayed the steepest decline, dropping below 0.5 within 45 min, while Ni‐MOF‐C followed a more gradual trajectory. Kinetic modeling using a pseudo‐first‐order approach (Figure 7e) yielded rate constants of 0.0157 and 0.0035 min^−1^ for Ni‐MOF‐A and Ni‐MOF‐C, respectively. The calculated rate constant for Ni‐MOF‐A was ≈4.5 times higher than that of Ni‐MOF‐C, confirming its better photocatalytic activity under the same conditions. The high correlation coefficients (R^2 ^= 0.99 for Ni‐MOF‐A and 0.98 for Ni‐MOF‐C) further support the applicability of the pseudo‐first‐order model in the 15–75 min interval. These values are consistent with the photodegradation efficiencies shown in Figure 7f. Ni‐MOF‐A achieved complete MO removal by 150 min, whereas Ni‐MOF‐C reached 82.2%. This high performance of Ni‐MOF‐A was achieved under mild conditions, using a low catalyst loading of 0.5 g L^−1^ and without any dopants, composite structures, or high‐energy light sources. To the best of our knowledge, this is the first direct comparison of amorphous and crystalline Ni‐MOFs in the context of MO photodegradation under UV light. The better performance of Ni‐MOF‐A is attributed to its amorphous structure, higher surface area (≈242 m^2 ^g^−1^), and larger pore volume, which likely facilitate improved dye adsorption and molecular diffusion compared to the more compact and crystalline Ni‐MOF‐C. Ni‐MOF‐C showed inferior photocatalytic performance despite its higher zeta potential. Since the suspensions were stirred during degradation, colloidal stability was not expected to be a limiting factor. This suggests that the better photocatalytic activity of Ni‐MOF‐A is primarily related to its higher surface area and porosity.

Many studies report the use of nickel‐based or bimetallic nickel containing MOFs for photocatalytic degradation, but most target pollutants are other than MO. Reported systems have focused on dyes such as rhodamine B,^[^ ^49^, ^50^ ^–^ ^51^ ^]^ congo red,^[^ ^50^ ^,^ ^51^ ^]^ methylene blue,^[^ ^52^ ^,^ ^53^ ^]^ and crystal violet,^[^ ^54^ ^]^ or compounds like tetracycline,^[^ ^55^ ^]^ and bisphenol A.^[^ ^56^ ^]^ These studies differ widely in light source, pollutant and catalyst loading, which limits the comparability of their photocatalytic performance with respect to MO. Nevertheless, a limited number of studies have specifically examined MO degradation using Ni‐MOFs, enabling a degree of comparison, although differences in synthesis complexity, light source, and catalyst formulation should be considered. For example, Ramezanalizadeh and Manteghi synthesized a Co/Ni‐MOF immobilized on magnetic BiFeO_3_, which achieved over 90% degradation of 50 mg L^−1^ MO in 90 min under 440 nm LED light, using a catalyst loading of 0.4 g L^−1^.^[^ ^57^ ^]^ Han et al. prepared a Ni‐ZIF/C_3_N_4_ composite via solvothermal synthesis, achieving 99.96% degradation of 10 mg L^−1^ MO in 180 min under a 150 W Xe lamp with *λ* > 420 nm, using 1.0 g L^−1^ catalyst.^[^ ^58^ ^]^ In contrast, the Ni‐MOF‐A and Ni‐MOF‐C used in this study were prepared via a simple one‐pot precipitation method in methanol at RT, without any post‐synthetic treatment or incorporation of additional components. This low‐energy, solvent‐friendly process shows a sustainable and scalable route to photocatalyst development. The high photocatalytic activity described by Han et al. was attributed to increased adsorption and improved charge separation in the composite system, which supports our interpretation that the improved photocatalytic behavior of Ni‐MOF‐A results from its large surface area and porous structure. Although their study did not directly investigate the contribution of reactive oxygen species (ROS) such as .OH, .O_2_^−^, or h^+^, their possible involvement was suggested. A similar ROS‐mediated mechanism may also occur in our system, as is often reported for MOF‐based photocatalysts activated by UV or visible light sources.^[^ ^59^, ^60^ ^–^ ^61^ ^]^ This possibility will be explored in future work.

## 3 Conclusion

Crystalline and amorphous Ni‐MOFs were prepared using a one‐pot method at RT in methanol with 2‐MIM as the organic linker. By varying the total solvent volume, two structurally distinct forms were obtained without the post‐synthetic modification. This approach represents an accessible and energy‐efficient route for tuning crystallinity in Ni‐MOFs under mild conditions. The comparison between the two phases revealed significant differences in structural, morphological and functional properties. The amorphous sample exhibited a higher surface area and pore volume, which correlated with superior gas sorption and photocatalytic degradation of MO. Crystalline MOFs are often favored for their well‐defined frameworks but this study demonstrates that amorphous analogs may outperform them in certain functional contexts, such as dye degradation and gas sorption. Future work could investigate other pollutants, evaluate photocatalytic activity in visible light, and investigate the reusability, electronic structure, and long‐term stability of these materials. Additional studies on the kinetics and selectivity of gas sorption would also help to clarify their potential for environmental applications.

## 4 Experimental Section

4.1

4.1.1

##### Materials

Nickel(II) nitrate hexahydrate (Ni(NO_3_)_2_·6H_2_O, 99.999%, trace metals basis) and 2‐methylimidazole (2‐MIM, ≥99%) were obtained from Sigma–Aldrich (Germany). MO was provided by Thermo Fisher Scientific (USA). Methanol and acetone were purchased from Lach‐Ner, s.r.o. (Czech Republic). All reagents were used as received without further purification.

##### Synthesis of Ni‐MOF

Both amorphous (Ni‐MOF‐A) and crystalline (Ni‐MOF‐C) Ni‐MOFs were synthesized via a one‐pot method at RT. For the preparation of Ni‐MOF‐A, 4.00 mmol of nickel(II) nitrate hexahydrate was dissolved in 50 mL of methanol, and 8.00 mmol of 2‐MIM was dissolved in a separate 50 mL of methanol. The two solutions were stirred individually and then combined by pouring the nickel solution into the ligand solution under continuous stirring. The mixture was stirred for 24 h at RT. The resulting precipitate was collected by centrifugation, washed several times with methanol, and dried in an oven at 80 °C overnight.

Ni‐MOF‐C was prepared using the same molar amounts of precursors, but with a reduced total solvent volume of 15 mL. Specifically, 4.00 mmol of nickel salt and 8.00 mmol of 2‐MIM were each dissolved in 7.5 mL of methanol. After mixing under identical conditions, the resulting precipitate was washed several times with acetone and dried as described above.

##### Characterization Techniques

Powder X‐ray diffraction (XRD) patterns of both Ni‐MOF‐A and Ni‐MOF‐C were recorded using an X’Pert MPD Pro diffractometer (PANalytical, Almelo, Netherlands) operating in Bragg–Brentano geometry with Cu K*α*_1,2_ radiation (*λ *= 1.5418 Å). The X‐ray tube was operated at 40 kV and 30 mA. An automatic divergence slit was used to maintain a constant irradiated area of ≈5 × 5 mm^2^ on the sample surface. Scattered X‐rays were detected using an X’Celerator position‐sensitive detector equipped with a *β*‐filter to suppress Cu K*β* radiation. The samples were mounted on non‐diffracting silicon substrates. Patterns were collected at RT over a 2*θ* range of 5–50°. Rietveld refinement of the XRD patterns was carried out using the WinPlotR/FullProf Suite software package.^[^ ^62^ ^]^ The instrumental resolution function (IRF) was determined from a standard LaB_6_ sample (SRM 660c, NIST) measured under identical conditions, and instrumental broadening was subtracted from the experimental data prior to analysis.

The morphology and elemental composition of the samples were analyzed using a scanning electron microscope SU5000 (Hitachi High‐Tech Corporation, Tokyo, Japan) operated at an accelerating voltage of 30 kV. The microscope was equipped with both secondary electron (SE) and backscattered electron (BSE) detectors and coupled with an EDAX Z2‐i7 EDX analyzer (AMETEK, Mahwah, NJ, USA). Samples were dispersed in acetone, drop‐cast onto silicon wafers, and dried at RT. The prepared wafers were mounted on aluminum stubs using carbon double‐sided adhesive tape. No conductive coating was applied prior to analysis.

Fourier‐transform infrared (FTIR) spectroscopy was used to identify functional groups and investigate bonding in Ni‐MOF‐A and Ni‐MOF‐C. Spectra were collected using a Nicolet iS50 spectrometer (Thermo Fisher Scientific, Waltham, MA, USA) equipped with a diamond attenuated total reflectance (ATR) module. Measurements were performed in the mid‐IR (4000–600 cm^−1^) and far‐IR (400–100 cm^−1^) regions, with a resolution of 4 cm^−1^ and 100 scans averaged per spectrum. Data were processed using the OMNIC software.

Dynamic light scattering (DLS) was employed to determine the particle size distribution and average hydrodynamic diameter of the samples using a Litesizer 500 instrument (Anton Paar, Graz, Austria). Measurements were conducted at RT using a semiconductor laser (40 mW, *λ *= 658 nm) in backscattering mode (175° detection angle). Prior to analysis, samples were dispersed in deionized water and sonicated for 10 min to ensure homogeneous suspensions. Each suspension (1 mL) was then transferred into a quartz cuvette and equilibrated for 2 min before measurement. Results were averaged from 5 individual measurements, with an experimental error of ≈5%.

Zeta potential measurements, conducted on the same instrument, assessed the surface charge and colloidal stability of the Ni‐MOF particles. Samples were dispersed in freshly purified deionized water (pH 5.8, Ewa 20 n purification system; Watek, Ledeč nad Sázavou, Czech Republic). For each measurement, 0.85 mL of sample suspension was transferred into an Omega cuvette. The zeta potential values were calculated using the Smoluchowski approximation. Results were averaged from 10 individual measurements, with an experimental error of ≈5%.

Specific surface areas and pore volumes of Ni‐MOF‐A and Ni‐MOF‐C were determined from nitrogen adsorption–desorption isotherms measured using a Nova 3200 sorption analyzer (Anton Paar, Graz, Austria). Prior to analysis, samples were degassed at RT for 24 h. Isotherms consisting of 60 adsorption and desorption points were obtained using nitrogen gas (Linde, 99.999%) at liquid nitrogen temperature (−196 °C). Specific surface areas were calculated using the 5–point Brunauer–Emmett–Teller (BET) model and the Micropore BET Assistant within the NovaWin software. Pore volumes were evaluated using the Barrett–Joyner‐Halenda (BJH) method. Each sample was measured 3 times, with a measurement uncertainty of ≈5%.

CO_2_ adsorption capacities were evaluated from adsorption isotherms obtained using an Autosorb iQ3 analyzer (Quantachrome Instruments, Boynton Beach, FL, USA). Prior to measurements, samples were degassed at RT for 24 h. CO_2_ adsorption–desorption isotherms were measured at 0 °C across a pressure range of 0–760 Torr using high‐purity CO_2_ gas (99.999%). Each measurement was repeated 3 times, with an experimental uncertainty of ≈5%. The maximum CO_2_ adsorption capacity was determined directly from adsorption isotherms using the Autosorb software.

The photocatalytic activity of Ni‐MOF‐A and Ni‐MOF‐C was evaluated by monitoring the degradation of MO under UV irradiation. The MO stock solution was prepared by dissolving 10 mg of dye in 1 L of distilled water, yielding an initial dye concentration of 10 mg L^−1^. In a typical photocatalytic experiment, 25 mg of catalyst (Ni‐MOF‐A or Ni‐MOF‐C) was dispersed into 50 mL of the MO solution in a glass beaker. The suspension was magnetically stirred in the dark for 1 h to achieve adsorption–desorption equilibrium. Subsequently, the mixture was irradiated using a 40 W UV lamp (*λ *= 365 nm) placed ≈8 cm above the solution surface. During irradiation, aliquots of 4 mL were collected at predefined intervals (0, 15, 30, 45, 60, 75, 90, 105, 120, 135, and 150 min), centrifuged to separate the catalyst, and 3 mL of the clear supernatant was analyzed using a Cary 300 UV–Vis spectrophotometer (Agilent Technologies, Santa Clara, CA, USA) in the wavelength range of 200–800 nm, monitoring specifically the absorbance maximum at ≈464 nm. A control experiment without catalyst was performed under identical conditions to assess dye stability under UV exposure.

The photocatalytic degradation of MO was analyzed assuming pseudo‐first‐order kinetics, following (Equation 2)

(2) $$\ln\;\left(\frac{A_{0}}{A}\right)=kt$$

where *A*_0_ and *A* represents the absorbance at 464 nm at time 0 and time *t*, and *k* is the apparent rate constant (min^−1^). This approach is based on the assumption that MO concentration is directly proportional to absorbance, in accordance with the Beer–Lambert law.

## Conflict of Interest

The authors declare no conflict of interest.

## Author Contributions

**Viktorie Neubertová**: conceptualization (lead); investigation (equal); methodology (equal); validation (equal); visualization (lead); writing—original draft (lead). **Jaroslava Jarolímková**: investigation (equal). **Stanislav Daniš**: investigation (equal). **Ľuboš Vrtoch**: investigation (supporting). **Zdeňka Kolská**: funding acquisition (lead); investigation (equal); supervision (lead).

## Data Availability

The raw data required to reproduce the above findings are available to download from Zenodo under the following DOI:10.5281/zenodo.15826760.
